# Clinical and neurophysiological study of peroneal nerve mononeuropathy after substantial weight loss in patients suffering from major depressive and schizophrenic disorder: Suggestions on patients' management

**DOI:** 10.1186/1749-7221-3-24

**Published:** 2008-11-12

**Authors:** Aikaterini Papagianni, Panagiotis Oulis, Thomas Zambelis, Panagiotis Kokotis, George C Koulouris, Nikos Karandreas

**Affiliations:** 1 Laboratory of Electromyography and Clinical Neurophysiology, Department of Neurology, Aeginition Hospital, Medical School, University of Athens, Greece; 2 Department of Psychiatry, Aeginition Hospital, Medical School, University of Athens, Greece

## Abstract

**Background:**

Peroneal nerve is susceptible to injuries due to its anatomical course. Excessive weight loss, which reduces the fatty cushion protecting the nerve, is considered a common underlying cause of peroneal palsy. Other predisposing factors, such as prolonged postures, traumas of the region or concomitant pathologies (for example diabetes mellitus) contribute to the nerve damage. This study aims to reveal the multiple predisposing factors of peroneal nerve mononeuropathy after substantial weight loss that coexist in psychiatric patients and to make suggestions on their management.

**Methods:**

Nine psychiatric inpatients, major depressive or schizophrenic, with foot drop underwent a complete clinical neurological and neurophysiological examination. All had excessive weight loss, which was completed in a short period of time and had not resulted from a well-balanced low-calorie diet, but was due to their psychiatric illness. Data regarding predisposing factors to peroneal nerve mononeuropathy were gathered, such as habitual leg crossing, squatting or other prolonged postures.

**Results:**

The clinical examination and the neurophysiological evaluation in all patients were indicative of a focal lesion of the peroneal nerve at the fibular head.

**Conclusion:**

Patients with major depressive and schizophrenic disorders gather multiple predisposing factors to peroneal palsy, adequate to classify them at a high risk group. The better focus of the attendant medical and nursing staff on this condition, the early clinical and neurophysiologic evaluation and surgical interventions may enable an improved management and prognosis of these patients.

## Background

It is well established that the peroneal nerve is susceptible to injuries due to its anatomical course. The common peroneal nerve (PN) originates as one of the two terminal divisions of the sciatic nerve at the apex of the popliteal fossa. At the level of the fibular head it divides into its terminal branches (deep and superficial PN). Due to its superficial anatomical course around the fibular neck, where it is covered only by skin, subcutaneous tissue and a fat pad, the nerve is susceptible to damage due to pressure against the bone [1].

The main presenting symptom in lesions of PN is footdrop, due to paresis of the dorsiflexor muscles of the foot and toes. When severe, it can be noticed as a change in the patient's gait (steppage gait) (i.e. the patient raises the foot higher, when swinging it forward, to avoid striking the toes on the ground). Sensory deficits, such as decreased touch and pin-prick sensation over the anterolateral leg and dorsum of the foot, are more common in such cases, rather than pain or paresthesias [1].

Excessive weight loss, which reduces the fatty cushion protecting the nerve, is considered one of the most frequent, if not the most common underlying cause of peroneal palsy [1,2]. Endocrine and metabolic disorders (i.e. diabetes mellitus, alcoholism, thyrotoxicosis or vitamin B depletion), trauma [3], perioperative damage [1,3], venous thrombosis [4], habitual leg-crossing and prolonged squatting have also been identified as predisposing factors [3]. The association between weight loss, leg crossing and the development of pressure paralysis of the peroneal nerve was first described in 1929 by Woltman H.W. [5].

Isolated peroneal palsy has been previously observed exclusively in patients suffering from depression [6-8]. In this study, we present nine psychiatric inpatients, suffering from major depressive or schizophrenic disorders who developed peroneal nerve mononeuropathy after substantial weight loss. This group of patients was studied clinically and neurophysiologically in an attempt to present the multiple predisposing factors that coexist in this category of patients and to make suggestions regarding their management.

## Methods

Nine Caucasian psychiatric inpatients (eight male) were referred to our laboratory from 1992 until 2008, because of footdrop. In one patient (case 9), footdrop was the secondary reference indication, since it occurred seven years prior to examination. The mean age of patients was 46.8 years (range 29–73 years). All had substantial weight loss, more than 10% of their initial body weight (in five patients the loss was greater than 20%). The weight loss occurred in a short period of time (weeks). Five patients suffered from a major depressive episode in the context of a major depressive disorder and four from a schizophrenic disorder, according to the DSM-IV diagnostic criteria [9]. This retrospective study was conducted according to the principles outlined in the Declaration of Helsinki.

Initially, the following clinical data were acquired: symptoms and duration, type of onset, psychiatric history, duration of illness, number of previous hospitalizations, medications, weight loss (in kilograms, percentage of initial weight and rate of loss), other predisposing factors (e.g. inactivity, habitual leg-crossing, squatting, prolonged postures, trauma), and concomitant pathology (diabetes, metabolic or toxic diseases, alcohol abuse, vitamin deficiency). Data on the concomitant pathology were additionally checked by a set of appropriate laboratory tests (Table 1).

**Table 1:** Clinical data

Patient	Sex	**Age **(years)	Psychiatric illness	Duration of psychiatric illness/number of previous hospitalizations	**Weight **(in kg)/loss (in kg)/% initial body weight	**Onset of symptoms **(in days prior to examination)	Main presenting symptom	Medications	*Concomitant pathology*
1	Male	57	Major depressive episode in the context of a major depressive disorder	7 months/2 hospitalizations.	71/12/16.9%	20	Footdrop R, weakness of dorsiflexor muscles R.	maprotiline amitriptyline, diazepam, levomepromazine	*none*
2	Male	43	Schizophrenic disorder	23 years/none	70/20/22.2%	45	Footdrop L	olanzapine, haloperidol, biperiden	*well controlled diabetes*
3	Male	29	Schizophrenic disorder	6 years/2 hospitalizations	68/20/22.7%	18	Weakness R	trifluoperazine, amitriptyline biperiden.	*none*
4	Male	62	Major depressive episode in the context of a major depressive disorder	1 year/1 hospitalization	67/18/21.2%	55	Weakness of dorsiflexor muscles L	amitriptyline, clomipramine, chlorpromazine, quazepam.	*none*
5	Male	36	Major depressive episode in the context of a major depressive disorder	4 years/none	92/23/20%	20	Weakness of dorsiflexor muscles L > R	clomipramine, sertraline	*none*
6	Female	73	Major depressive episode in the context of a major depressive disorder	2 years/1 hospitalization	66/10/13.2%	45	Weakness of dorsiflexor muscles R	paroxetine, mirtazapine	*none*
7	Male	38	Schizophrenic disorder	2 years/2 hospitalizations	70/13/15.6%	60	Footdrop L, weakness of dorsiflexor muscles L.	aripiprazole, olanzapine	*none*
8	Male	41	Schizophrenic disorder	21 years/3 hospitalizations	118/35/22.9%	7 years (2520)	sensory deficits R	risperidone, clomipramine, bromazepam	*well controlled diabetes*
9	Male	43	Major depressive episode in the context of a major depressive disorder	20 years/1 hospitalization	106/12/11.3%	90	Footdrop R, weakness of dorsiflexor muscles R	venlafaxine hydrochloride, levomepromazine, quetiapine fumarate, escitalopram, clorazepate dipotassium, lamotrigine	none

All patients underwent a complete clinical, neurological examination. Especially, the bulk and contour of tibiofibular muscles were examined and the muscle strength of posterior thigh and tibiofibular muscles was graded according to the British Medical Research Scale. Light touch and pinprick sensation were tested in the cutaneous distribution of the lateral cutaneous nerve of the calf and in the superficial and deep sensory branches of the common peroneal nerve. Polyneuropathy was assessed with NSS (Neurological Symptom Score) [10].

The neurophysiological examination was conducted using standard methods in a warm room, with a Nihon Kohden Neuropack Σ. Motor and sensory nerve conduction studies of peripheral nerves in the lower (deep peroneal, tibial, superficial peroneal and sural nerve) and upper limbs (median and ulnar nerve) as well as concentric needle electromyography of muscles in the lower limbs were performed. In particular, the motor conduction velocity of the peroneal nerve at the ankle, below the fibular head and at the lateral popliteal fossa and the sensory conduction velocities of the superficial peroneal and the sural nerve were calculated. The electromyographic evaluation of the tibialis anterior, the extensor digitorum brevis, the peroneus longus and the gastrocnemius was performed using a disposable concentric needle electrode (Medtronic DCN 50), at rest and during voluntary action.

## Results

The clinical examination and the neurophysiological evaluation in all patients were indicative of isolated damage of the peroneal nerve. None of the patients had abnormalities in the distribution of other peripheral nerve of the limb, indication of lumbosacral radiculopathy, plexopathy, or sciatic neuropathy. Moreover, none met the criteria for a generalized peripheral neuropathy. Six patients did not have concomitant pathology nor did they receive medications known to cause peripheral neuropathy. Two patients suffered from a well-controlled type 2 diabetes mellitus, according to laboratory findings.

The electrophysiological studies of the deep peroneal nerve showed a partial motor nerve conduction block, according to the AAEM criteria [11], at the fibular head in seven patients (cases 1–5, 7, 9), and slowing of the conduction velocity in the popliteal fossa – fibular head segment in six (cases 2–5, 9). In cases 6 and 8, the findings were indicative of axonal damage. The amplitude of the sensory potential of the superficial peroneal nerve was abnormally low in five patients, while sensory conduction velocity was within normal limits in all but one patient (case 9). (Table 2)

**Table 2:** Motor and Sensory (antidromic method) nerve conduction study of the peroneal nerve, using superficial recording electrodes

Patient	**MCV^1 ^Popliteal Fossa- fibular head **(m/sec)		**MCV Below fibular head- ankle **(m/sec)		**CMAP^2 ^Amplitude Popliteal Fossa **(mV)		**CMAP Amplitude Ankle **(mV)		**Distal CMAP Latency **(ms)		**SCV**^3 ^(m/sec)		**SNAP^4 ^Amplitude **(μV)		**Distal SNAP latency **(ms)	
	**L**	**R**	**L**	**R**	**L**	**R**	**L**	**R**	**L**	**R**	**L**	**R**	**L**	**R**	**L**	**R**

**1**	48	46	48	46	4.0	1.0	4.0	3.0	4.0	4.2	45	44	7	5	2.9	3.1
**2**	32	48	47	49	0.5	4.3	2.5	5.0	3.6	4.0	51	52	4	16	2.9	2.5
**3**	46	35	45	37	6	0.5	7.0	6.0	4.7	4.7	50	45	30	15	2.7	3
**4**	32	49	50	52	0.2	4	2.5	3.0	4.1	4.8	50	50	6	6	2.4	2.5
**5**	14	25	39	42	0.5	0.5	4.0	5.0	4.5	4.0	40	40	4	5	3.7	3.1
**6**	54	53	53	51	7.0	2.0	7.0	2.4	3.5	4.0	51	50	14	12	2.8	2.6
**7**	46	46	46	44	0.8	6.0	2.0	6.0	4.0	4.0	46	50	5	11	2.8	2.6
**8**	53	45	50	47	6.0	2.5	6.0	2.3	3.9	4.5	55	57	9	8	2.4	2.6
**9**	50	35	48	46	5	1	6	5	4	4.2	50	44	9	4	2.8	3.6

^1 ^Lower normal limit 42 m/sec. The lower normal limit of MCV and SCV indicative of axonal damage is 29.5 m/s

^2 ^Lower normal limit 3 mV

^3 ^Lower normal limit 42 m/sec

^4 ^Lower normal limit 5 μV

The electromyographic evaluation in nine patients showed a reduction in the number of recruited motor units of the muscles innervated by the peroneal nerve. In six patients (cases 1, 2, 3, 5, 7, 9) there were also signs of ongoing denervation (fibrillation potentials and/or positive sharp waves). In case 9, the data were indicative of reinnervation process (no spontaneous activity, polyphasic, but stable motor unit action potentials).

The above are suggestive of a focal lesion of the peroneal nerve at fibular head, which consisted in demyelination with conduction block in seven patients and concomitant axonal loss in five. The lesion was predominantly axonal in patients 6 and 8 and there were findings of residual axonal damage in patient 9.

## Discussion

Although, peroneal nerve palsy is a common mononeuropathy (approximately 15% of all mononeuropathies in adults) [2], few reports have previously been published regarding patients with psychiatric history. Moreover, all of these studies regard, exclusively, suffers from depression. Our group of patients comprises both sufferers of depression and schizophrenia. All had excessive weight loss which, noticeably, was completed in a short period of time and did not result from a well-balanced low-calorie diet, but was due to their psychiatric illness. Appetite loss is common in major depression. Delusional beliefs of worthlessness, guilt and deserved punishment leading to food abstinence are also common. Delusional beliefs of persecution (e.g. food poisoning) or of religious content with fasting are not infrequent in schizophrenic patients. It is plausible that our patients were deficient in certain vitamins or other nutrients necessary for nerve function, though no relevant data were gathered. Weight loss is highlighted as an important factor in this study, since several components of the patients' psychopharmacological regimen have as a side-effect weight gain (e.g., amitriptiline, chlomipramine, olanzapine) [12]. The patients tended to take prolonged postures, such as leg-crossing or squatting, or displayed immobility. The above can be observed commonly both in patients with major depression and chronic schizophrenia.

The neurophysiological examination of our patients was suggestive of an entrapment neuropathy of the peroneal nerve at the fibular head. The conduction studies and electromyography showed that the underlying pathology was focal demyelination presenting as conduction block, with or without significant reduction of conduction velocity and a varying amount of axonal damage. In case 9, with a seven year history of peroneal nerve palsy, the findings were indicative of residual axonal damage. The involvement of a considerable proportion of axons is associated with a less favorable outcome and a prolongation of the rehabilitation time.

The study of this group of patients highlights matters regarding their management. (Table 3). Depressive and schizophrenic patients gather multiple predisposing factors to peroneal neuropathy. Weight loss in combination with psychomotor retardation, prolonged postures and inactivity are such factors placing these patients at a high risk group for peroneal palsy. This has already been suggested for patients with depression [7] but not, as yet, for schizophrenic patients.

**Table 3:** Suggestions on patients' management

First Evaluation	After the establishment of the diagnosis of peroneal nerve mononeuropathy	Preventive means
Detailed history; overcome obstacles in communication (onset of symptoms, weight loss, tendency to retain prolonged postures, e.g. squatting, legs crossed, is the patient bed-bound)	*If exclusive or predominant demyelination:* Conservative treatment (appropriate diet, mobilization physiotherapy)	Information of medical and nursing staff in psychiatric units
Complete clinical neurological examination	*If predominantly axonal lesion and/or anatomical causes:* Reference to qualifying centre for surgical repair (e.g. neurolysis) Physiotherapy (approximately 3 weeks after the surgery) Clinical outcome evaluation-follow up after 6 months If not satisfied with the clinical outcome, consideration for additional surgical management (e.g. tendon transfer)	Patients' weight monitoring, establishment of well-balanced, nutritious dietary plan
Reference for neurophysiological and electromyographic examination		Mobilization of patients and avoidance of prolonged postures

In order to provide the best possible management, several obstacles to communication should be overcome. These patients might not complain of their symptoms or, conversely, might exaggerate their complaints, making them less believable. Moreover, questions about whether the patients' symptoms are genuine or delusional, or hypochondrial, can only be answered by a thorough clinical and, eventually an electrophysiological examination. In addition to the above, an MRI of the lower leg region should be considered, in order to exclude other lesions causing peroneal nerve damage (e.g. ganglion cyst, aneurysm, synovial cyst or osteochondroma) [13].

Although most peroneal palsies due to demyelination recover spontaneously after removal of the predisposing factors, there are a number of cases, exemplified by patient 9, where concomitant axonal damage can lead to prolonged or permanent paralysis and atrophy and thus need to be evaluated for an eventual surgical intervention. The neurophysiological examination can establish the existence and degree of axonal involvement, providing a basic indication for interventional treatment. The time course of the repair interventions is an important factor. Patients should be referred as soon as possible to a qualified centre, where restoration techniques of the nerve function can be performed. The surgical repair is the usual management, at least initially. Surgical exploration, neurolysis, partial fibulectomy and graft repairs (from the sural or tibial nerve) are commonly performed [14,15]. If nerve surgery fails to reconstitute a useful foot lift, patients need to be evaluated for their suitability to undergo tendon transfer or other reconstructive procedures. In about 3 weeks after the surgery, the patients can begin physiotherapy. It is advisable that a follow-up with clinical and neurophysiological examination at 6 months should be programmed, in order to reveal the degree of function recovery.

As far as prevention is concerned, the role of the attendant medical and nursing staff is important. A well-balanced and nutritious dietary plan should be established for these patients, who should be also instructed to avoid habitual, prolonged postures during which pressure is exerted on the nerve.

## Conclusion

The findings of this study suggest that more attention should be given to identify early signs of peroneal neuropathy in psychiatric inpatients with major depressive or schizophrenic disorders. Prompt neurological and neurophysiological examination can reveal the extent and the severity of the damage and to enable early surgical interventions, contributing to the better management and long-term prognosis for these patients.

## Competing interests

The authors declare that they have no competing interests.

## Authors' contributions

NK proposed the initial design of the study and supervised the preparation of this manuscript. NK, TZ, and PK performed the examinations on the patients. AP and GCK reviewed the laboratory records and the patients' history. AP prepared the initial and the revised draft of the manuscript, which was edited according to the propositions of all authors. Comments on the psychiatric aspects of this study were made by PO, who also helped to draft the manuscript. All authors read and approved the final manuscript.
